# Addressing the social determinants of youth mental health in Newham and Northern Devon: reflections on co-design and systems change

**DOI:** 10.1186/s12889-026-26586-z

**Published:** 2026-02-20

**Authors:** Ediane Santana de Lima, Nirandeep Rehill, Katie Potter, Cristina Preece, George Davis, Sophie Bulmer, Kate Allen, Anna March, Tim Hobbs, Peter Fonagy

**Affiliations:** 1 Dartington Service Design Lab, Bristol, UK; 2 UCLPartners, London, UK; 3 Dartington Service Design Lab, Stoke Gabriel, UK; 4 Dartington Service Design Lab, Hereford, UK; 5 Dartington Service Design Lab, London, UK; 6 University of Exeter, Exeter, UK; 7 Dartington Service Design Lab, University College London, Totnes, UK; 8 Division of Psychology and Language Sciences, University College London (UCL), London, UK; 9 Menninger Department of Psychiatry and Behavioural Sciences, Baylor College of Medicine, London, UK

**Keywords:** Adolescent mental health, Systems change, Place-based co-design, Community-based participatory research, Group model building, Systems mapping, Social determinants, Participatory design

## Abstract

**Background:**

Despite increasing efforts to provide support for children and young people’s mental health and wellbeing, rates of poor wellbeing and probable mental health disorders continue to rise. This underscores the need for approaches that target the root causes and social determinants underlying these issues. This paper offers reflections on efforts to address these determinants at the local level, drawing on learning from the co-design phase of Kailo, a research and design initiative that works with young people and communities to shift the underlying conditions sustaining poor mental health and wellbeing, including resources, policies, relationships, and mindsets.

**Methods:**

Across two pilot sites, Northern Devon and Newham, the Kailo programme used a community-based co-design methodology and a structured process for reflective, iterative learning. Participants included young people, community partner organisations, local professionals, academics, researchers, and designers. The co-design process was integrated with research synthesis and systems mapping to ensure that emerging strategies were informed by lived experience, empirical evidence, and a systemic lens.

**Results:**

Four co-design strategies were developed through this process, involving over 300 young people and community members across the two pilot sites. While the intention was to support upstream prevention and systems-level transformation, most strategies ultimately focused on individual-level interventions. Nonetheless, the process generated important learning about the barriers and enablers to developing systems change approaches targeting social determinants and preventative action.

**Conclusion:**

Systemic co-design offers potential for developing youth-centred, locally situated policy and practice interventions. However, careful attention must be paid to the composition of co-design teams and in relation to who is included, who is more broadly engaged, and who is kept informed. This is particularly crucial when exploring system-wide solutions, rather than focusing solely on the immediate experiences of participants. While the significance of addressing social determinants was acknowledged and explored during the initial phases of problem identification and understanding, effective action in this area requires sustained collaboration, not only with young people, but also with other community members who hold valuable knowledge and experience regarding the use of, and opportunities within, systemic levers necessary to support transformative change.

## Background

Despite increasing efforts to provide mental health and wellbeing support for children and young people (CYP), reported rates of poor wellbeing and probable mental disorders have continued to rise [1, 2]. Demand for specialist services has also increased over the past decade [3]. Mental health specialists offer various explanations for this trend, including an increase in experienced symptoms of poor mental health [2] and the increased complexity of CYP’s lives. This includes new challenges associated with social media [4], uncertainty about the future [5], and environmental concerns [6]. While there is substantial evidence supporting these explanations, some commentators have also pointed to other contributing factors, such as changes in diagnostic criteria, i.e., *diagnostic inflation* [7], greater awareness, recognition, and possible over-interpretation of symptoms [1].

There is growing recognition of the importance of prevention and of broader influences beyond individual behaviour or genetic inheritance [8]. Recent approaches aim not only to respond to presenting symptoms such as distress or difficulty [9] but also to reduce risk factors and enhance protective ones [10]. Going further, Marmot et al. [11–13] have highlighted the need to tackle structural and contextual factors that perpetuate poor outcomes and drive health inequalities, in order to shape population mental health. The literature on both social determinants and systems change provides valuable conceptual frameworks, tools, and entry points for exploring potential interventions [14–16].

### Social determinants

Research on social determinants explores how social, environmental, and behavioural risk factors [17, 18] shape mental health and wellbeing outcomes [19]. To comprehensively support CYP, it is necessary not only to offer targeted and specialist services for those with diagnosable conditions and to invest in early intervention and health promotion, but also to address these foundational social determinants [9, 20]. Adopting a more universal and holistic approach that centres on the wider challenges CYP face allows for improvements not only in mental health but also in broader life outcomes [21, 22].

It has been argued that greater attention is needed to the social and physical environments in which CYP live, and how these shape their exposure to the social determinants of health inequality [23]. These experiences can vary significantly across local contexts [24, 25]. For example, CYP in both rural and urban settings may be exposed to determinants such as poverty, racism, or limited employment opportunities, but the ways these factors manifest are highly context dependent. In rural areas, job insecurity may reflect limited industry diversity and poor transport infrastructure [26, 27]. In contrast, urban areas often experience higher concentrations of poverty and job competition, particularly for entry-level or low-skilled roles, due to large numbers of young jobseekers [28]. Overlooking contextual factors, such as socio-economic influences, variable and shifting demographics, history and culture in a place can lead to an underdeveloped understanding of needs and local influences [25]. This lack of understanding can undermine the design and implementation of suitably nuanced and contextualised strategies to improve young people’s mental health and wellbeing [29].

### Systems change

The systems change literature offers additional concepts and frameworks for understanding the complex and structural roots of mental health challenges and their associated social determinants [30]. It refers to interventions that target the drivers of complex or *wicked problems* [31–33], rather than addressing individual-level symptoms, needs, or isolated risk and protective factors [22]. Drawing on the Social Innovation Generation (SIG) definition, Kania, Kramer and Senge [34] argue that systems change involves shifting the conditions that sustain problems, including policies, practices, resource flows, relationships, power dynamics, and mental models. It also entails developing initiatives that are sustainable over time [35]. While not a new concept, systems change has been inconsistently articulated and is often difficult to implement in practice [34, 36, 37].

A core feature of many systems change frameworks is the centrality of lived experience, particularly the involvement of those most affected by the systems in question [38, 39]. In the context of youth mental health, this underscores the importance of meaningfully engaging CYP and other community members in exploring, co-designing, and implementing strategies to support mental health and wellbeing, particularly those aiming to work systemically and sustainably [40]. Understanding how social determinants are experienced in different local contexts can help tailor interventions to be more effective and relevant [14].

Involving CYP in participatory methods such as co-design can not only enhance the relevance of strategies but can also offer direct wellbeing and empowerment benefits to the CYP involved, [41]. Benefits extend to broader peer groups through application of their insights [42]. However, the evidence base around how co-design works, and its impact on CYP mental health outcomes, remains underdeveloped [43]. Further research is needed to understand the conditions under which co-design is most effective [44, 45]. To our knowledge, no studies have examined how co-design can be utilised alongside systems change in youth mental health prevention.

### Focus of this paper

The aim of this paper is to present the challenges and opportunities of using co-design, informed by systems change, to address social determinants of youth mental health at a local level. The learning was generated as part of the Kailo research programme [46]. Recommendations support the ongoing development of the Kailo approach but are also relevant to other local systems change initiatives and may contribute to the evaluation of similar co-design efforts.

### Context: about Kailo

Kailo is a place-based research and design initiative that supports local partnerships to identify priorities and co-design, implement, and embed systemic strategies to address the social determinants of CYP’s mental health and wellbeing [46, 47]. The ultimate aim is to develop and test a framework that enables these local partnerships to do this work independently.

The programme is funded by the UK Prevention Research Partnership and brings together interdisciplinary expertise in adolescent mental health, design, research and systems thinking.

**Fig. 1:**
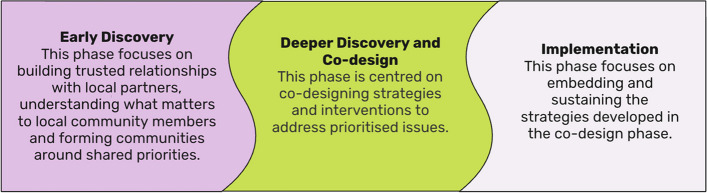
Kailo programme phases

Kailo unfolds over three broad phases (Fig. 1 adapted from Santana de Lima et al., [48]):

This paper focuses on insights generated from the Deeper Discovery and Co-Design phase of Kailo (Fig. 1). This phase sought to co-design local strategies addressing social determinants of young people’s mental health and wellbeing in two pilot areas: a rural/coastal area in Southwest England (Northern Devon) and an ethnically diverse and densely populated inner-city area of East London (Newham). The specific issues to be addressed were collaboratively identified and prioritised by CYP, community partners and system leaders during the preceding Early Discovery phase. These were framed as ‘Opportunity Areas’ (OAs) [26]. In Northern Devon, prioritised OAs were: (i) building stronger informal community support networks to promote mental health awareness and literacy (OA1); and (ii) creating and enhancing access to more diverse opportunities for studies, employment, and recreation (OA2). A third OA (iii) fostering a sense of identity and belonging was initially prioritised but later reframed as a cross-cutting theme as it overlapped with both OA1 and OA2. In Newham prioritised OAs were: (i) reducing the impact of violence and crime on young people’s mental health (OA3); and (ii) strengthening the role of local community infrastructure and activities for wellbeing (OA4) [48].

## Methods

**Fig. 2:**
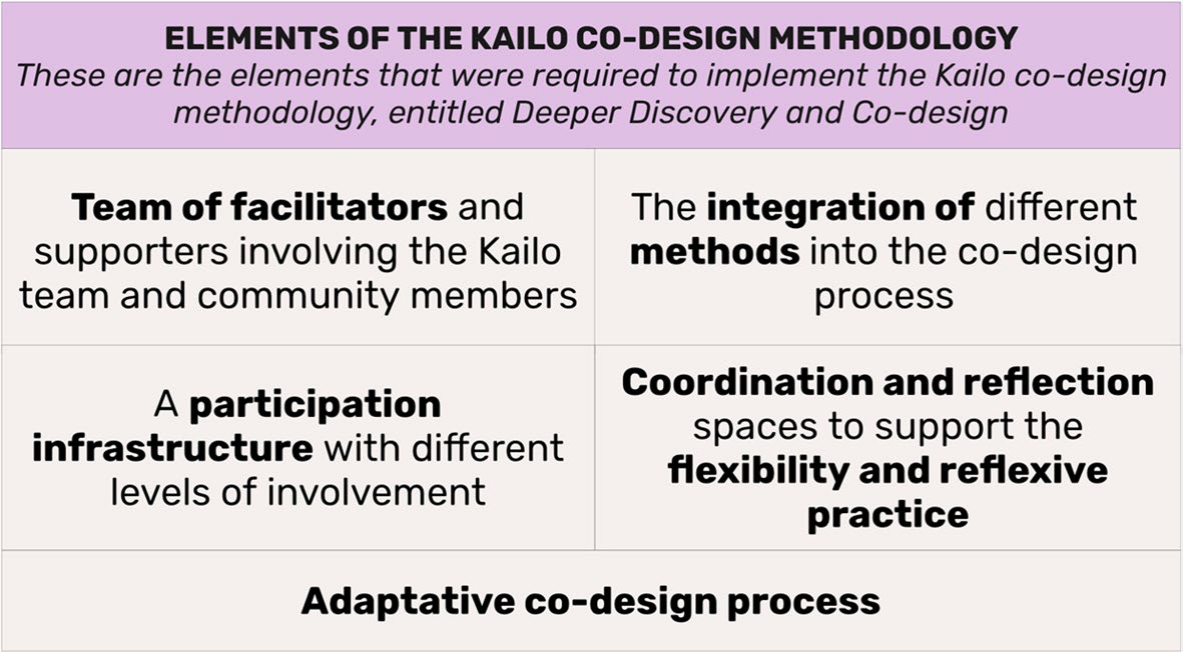
Core elements that are required to implement the Kailo co-design methodology

To address each opportunity area, Deeper Discovery and Co-design involved 16–19 workshops per opportunity area, and several ad hoc community engagements. The methodology for the *Deeper Discovery & Co-design* phase is structured around five core elements (see Fig. 2):

### Core team: facilitation and support

Each Kailo pilot site (i.e., Newham and Northern Devon) had two co-design delivery teams integrating research, design and youth work (Table 1). Individuals fulfilled Kailo research and design functions from a range of job roles. Community researchers – young people from Newham and North Devon, in an employed role—were integral to teams, for example, through designing engaging activities, facilitating sessions and synthesising session outputs. Community organisation members brought youth work skills to support session facilitation, co-designer recruitment, strategic direction, ongoing reflection and pastoral support to the CYP involved in the co-design process [48].

**Table 1:** Co-design delivery team composition for the two Kailo pilot sites

Newham	Northern Devon
Research & design team • Three Implementation specialists • One Public health specialist • Two community researchers – young people living in the pilot sites with experience of the prioritised Opportunity Areas (OA) • One Systems modeller Community Partners • Representatives from five Community organisations involved in youth and community work and support related to the prioritised OA themes (e.g., youth workers)	Research & design team • One Designer • One Researcher • Two community researchers – young people living in the pilot sites with experience of the prioritised Opportunity Areas (OA) • One Systems modeller Community Partners • Representatives from six Community organisations involved in youth and community work and support related to the prioritised OA themes (e.g., youth workers, mental health professionals, neurodiversity advocates)

### Integration of research & design components

To maximise the benefits of co-design and ensure structural issues were considered using multiple sources of evidence, two research methods were incorporated into the process. Early sessions used Group Model Building (GMB), a system dynamics participatory approach which enables the exploration of complex problems through mapping and facilitation of consensus building around a common view of a system [49]. Research evidence summaries, to provide the evidence base on what may be harmful, ineffective, impactful, or adapted for different contexts [50], were planned across sites through evidence briefings and planned evidence reviews. Implementation is discussed in the results and expanded in an accompanying paper [48] 

#### Participation infrastructure.

Delivery teams sought to develop a process that enabled meaningful participation from a range of community members in the co-design process, while centring the lived experiences of CYP in the two pilot sites. The approach was informed by co-design principles outlined in McKercher’s Beyond Sticky Notes [51], particularly the concept of “Small Circles”, in which people come together to build trust and initiate change starting from their own experiences. This was aligned with key frameworks for centring CYP in participatory processes, including Hart’s Ladder of Children’s Participation [52] and the Lundy Model [53].

The Deeper Discovery Circles were designed to operationalise both McKercher’s Small Circles [51] and the shared decision-making spaces described by Hart [52] and Lundy [53]. Importantly, the Lundy Model emphasises that creating a space for CYP to express their views is insufficient on its own; their views must also have an audience and exert influence [53]. The wider circles described below aim to reflect these requirements (Table 2).

**Table 2:** Overview of deeper discovery and co-design circles [48]

Group	Participants	Purpose and Role	Approach to Participant Engagement
Small Circle of Co-Design	Local CYP, and parents/carers or other community members (some offering support to enable meaningful CYP participation)	Lead the co-design process by generating ideas and responses to the OAs that are locally and contextually relevant. Intended as a reliable and trusted space where change can be imagined and created [51]	Fortnightly in-person co-design sessions
Circle of Research	CYP, community members, local organisations	Provide complementary community insights to inform strategy development in the Small Circles. The direction and focus were led by outputs and discussions emerging from the Small Circle	Targeted methods such as surveys and additional engagements
Big Circle	Local stakeholders, including practitioners working with CYP, and policy or commissioning leads	Act as champions for Kailo within local systems, offering ongoing review and feedback, linking strategies to wider initiatives, and providing resources. During implementation, they help mobilise resources and sustain change [54]	Meetings and informal conversations. Engagement also included integration with existing community platforms and less targeted communication through established local networks

Table 2 is adapted from Santana de Lima et al. [48]

#### Participants in the deeper discovery and co-design phase

##### Sampling and recruitment

The Small Circles aimed to comprise CYP and community members most affected by the prioritised OAs, including groups identified as underrepresented in the previous Kailo programme phase. Recruitment was led by Community Partners, supported by Kailo teams, through an equity-focused strategy emphasising choice and safeguarding [48]. Using relationship-based sampling, community organisations supported the Kailo team in recruiting CYP involved in youth work activities and others in the community who would benefit from opportunities such as this, but did not have access, based on limited resources to support their participation. Using the same approach, adults involved in the Early Discovery phase, including youth workers and parents, were invited to participate in the Small Circles [48].

The Circle of Research included broader community members who were not part of the Small or Big Circles but contributed via online surveys. They were engaged through online platforms and included CYP and community members living in the Kailo pilot sites [48].

The Big Circle was composed of local system leaders, practitioners, and community partners, recruited from those who participated in Early Discovery and via snowballing based on stakeholder recommendations [48].

### Co-design process and analysis

#### Settings and delivery

Small circles were delivered in person within youth and community settings in Newham and Northern Devon. Each Small Circle consisted of sixteen sessions, held weekly or fortnightly, delivered by the co-design team with contributions from community members and CYP. As part of the Circle of Research, online surveys were disseminated to community members [48].

Big Circle sessions were delivered online, with additional ad hoc engagements conducted both online and in person. These sessions were led by Kailo researchers and facilitators, with support from community partners. Three online workshop sessions were facilitated in Northern Devon and one in Newham. In both sites, one-to-one meetings were offered to some individuals. Additionally, members of the Kailo team participated in local forums to share emerging findings and questions from Small Circles, and to explore resources and other implementation requirements [48].

#### Codesign process

Co-design processes require an iterative approach, offering co-designers varied participation options and session formats (e.g., activities involving drawing, painting, writing and speaking, individually, in small and big groups), to accommodate differences in group dynamics and individual participant needs [42, 55, 56]. Table 3 provides an overview of Kailo’s Deeper Discovery and Co-Design activities. Within each session type, there was flexibility to vary content, pace, structure and scheduling of individual sessions to support an inclusive and responsive process while maintaining progress toward shared objectives across all co-design circles [48].

**Table 3:** Overview of the co-design activities within Kailo’s Deeper Discovery phase

Session Number	Broad focus and methods	Aims and objectives of the methods and approaches
Prep sessions 1 and 2	• Small circle individual 121 s • CYP induction to the group space	• Setting the space • Building relationships • Creating safe spaces
Session 1, 2	• Small Circle aims • Group agreements and rules of engagement [57] • Roles and responsibilities • Hopes and Fears [58]	
Sessions 3,4,5	• Presentation of Opportunities areas prioritised in Early Discovery • Group Model Building sessions [59]: Exploration of trends over time Connection circles [60] • Big Circle input on maps developed through Group Model Building • Inputs from research evidence	• Mapping the system associated with the OAs • Understanding structural influences • Building Casual Loop Diagrams [61]
Session 6	• Considering places to intervene [62]	• Identifying places to design change
Session 7	• Reflections on process and outputs • Completion of research briefings for two Small Circles	• Reflecting on sessions process, supports and content to date
Sessions 8, 9	• Persona development and connection to social determinants [63, 64] • Experience mapping, • Prioritisation around systems map • Input from Circle of Research (surveys) to determine priorities • Big Circle inputs on priorities and survey results	• Further prioritisation • Identification of key stakeholders • Introducing members
Sessions 10, 11	• Experience mapping • Relationship building and creating safe spaces and group agreements with new members • Vision and journey mapping based on this	• Building relationships with new members • Creating safe spaces • Bringing co-design groups together to share learning across them (Northern Devon)
Session 12	• Building shared understanding with professionals that joined Small Circles	• Envisioning new futures [65]
Session 13	• Journey mapping and visioning [66] • Goals and vision/generating designs • Big Circle input into future vision • Inputs from research evidence	• Co-designing change
Session 14	• Blueprint development [67]	
Session 15	• Refining of co-design blueprints	
Session 16	• Session to prepare for celebration event • Reflections on the process and outputs • Celebration and sharing learning with wider community members, including Big Circle members	• Celebrating outputs • Sharing learning and considering next steps

Whilst there was variation across sites, and the circles within them, this is a broad representation of the activities in the Deeper Discovery and Co-design phase [48]

Adapted from Santana de Lima et al., [48]

For example, session times were adjusted at different points in response to participants’ safeguarding concerns, seasonal daylight changes, and competing commitments such as exams, work, and Ramadan. To address barriers related to transport, particularly for CYP in rural areas or those uncomfortable travelling after dark, taxis were provided and sessions were scheduled earlier. Emotional and mental health needs were supported through the presence of trusted adults and mental health professionals, alongside access to quiet rooms for participants who needed to step away. The content and facilitation approach were also adapted to enhance accessibility, including the use of a blend of individual and group activities to meet diverse individual needs and create a neuro-affirmative space [48].

### Co-design data and analysis

A range of data was collected across the Circles, including photographs of activity notes and other physical outputs, field notes from research and evaluation team members, meeting and whiteboard notes from Big Circle sessions, survey responses, and session recordings. These data were synthesised by the delivery teams and, where appropriate, collaboratively analysed within the Deeper Discovery Circles. Synthesised findings were then returned to the Small Circles, where local priorities were refined and outputs further developed [48].

### Reflection spaces to support ongoing learning and reflection

As participation is influenced by shifting perspectives, roles, and power dynamics, continual reflection supported teams in considering what was happening, what might require adjustment, and how different experiences could be integrated [68, 69]. Flexible facilitation and adaptive approaches were used to support equitable participation and to allow ongoing refinement of the framework. Site teams (comprising of co-design session facilitators, support staff, evaluation team members, community partners, and co-designers) held regular learning and reflection sessions, aimed to support the synthesis of insights across sites, share learning, identify where adaptations were needed to facilitate the ongoing work, and ensure that the data analysis reflected co-designers perspectives [48] (Table 4).

**Table 4:** Overview of the embedded learning and reflection sessions across the two pilot sites

Session	Details	Participants
Northern Devon/Newham internal team sessions	Weekly meetings to review outputs, share learning, and plan upcoming co-design sessions. This included reflection on facilitation approaches and analysis of session outputs	Site team (including facilitators and Community Researchers) and Evaluation team members
Work Stream 1 Sessions	Weekly, then fortnightly cross-site meetings to discuss learning, ensure alignment with Kailo’s aims, and manage logistical and operational planning	Programme Co-Director, Programme Coordinator and Insights Lead, Site team members from both sites, Evaluation team members
Community Partner sessions (Northern Devon only)	Monthly meetings to review session outputs and survey findings. Also used to discuss how to ensure co-design sessions remained safe spaces for young participants	Site team members and Community Partners
Research integration sessions	Initially fortnightly meetings to align research evidence with the co-design process	Programme Coordinator and Insights Lead, Site Team Leads, Site Systems Experts, Researchers (including Anna Freud Centre researchers)
Safeguarding sessions	Quarterly meetings to reflect on safeguarding and emotional wellbeing concerns arising in the co-design sessions	Safeguarding Lead, Programme Coordinator, Insights Lead, Site Leads

#### Reflexivity

The Kailo programme brought together diverse expertise in adolescent mental health, research, design, and systems thinking through co-design delivery teams (Table 1) and an interdisciplinary wider research consortium of senior academics, public health experts, and complexity specialists. These groups entered the programme with different priorities and interests, requiring ongoing negotiation, adaptation, and consensus-building. As a guiding principle to navigate these discussions throughout the Deeper Discovery process, the views and perspectives of those participating in the Deeper Discovery and Co-design Circles, and primarily those of the Small Circles were prioritised.

### Ethics

This research and design programme, including its co-design methodologies, received ethical approval from the University College London Research Ethics Committee (Reference: Project ID/Title 18,773/002). Key considerations in the ethics process included the requirements to facilitate sessions with young people across different age groups and involving adults. This was related to concerns around power differentials and exposure of younger participants to harmful content or behaviours. Group agreements were developed to ensure collective responsibility, and a robust safeguarding structure was implemented to ensure psychological distress and safeguarding issues could be appropriately monitored and escalated [48].

## Results

This section summarises participation, process integration and co-design outputs. We also examine the extent to which these strategies remained aligned with the overarching aim of co-creating responses to address the social determinants of young people’s mental health and wellbeing.

### Implementation: participants and integration

#### Participants

An overview of participants involved across each of the Deeper Discovery and Co-Design Circles is provided in Tables 5 and 6. Further details on participant backgrounds, diversity, and retention are available in the forthcoming methods paper [48].

**Table 5:** Participants in small circles

Site		Young People				Community Partners and Professionals	
		Age range (years) of young people attending sessions	Number attending at least one session	Number attending at least half of all sessions	Average (median) number of young people per session	Number attending at least one session	Average (median) number of community partners per session
Northern Devon	Group 1	12–22	13	13	10	9	3
	Group 2	15–25	8	6	5	12*	4
Newham	Group 1	14–17	14	9	8	3	1
	Group 2	16–26	25**	8	8	6	3

^*^Northern Devon Group 2: The high number of community partners and professionals reflects an expansion of an existing group, with additional professionals joining over time rather than a high turnover

^**^Newham Young People: Attendance figures showed that although many young people engaged initially, fewer sustained participation, requiring ongoing recruitment to maintain session numbers (hence why the number of young people attending at least one session is high compared to the number attending at least half of the sessions). This was related to CYP life transitions and addressed through adaptation of session times and more focused recruitment [48]

**Table 6:** Participants in the big circle and circle of research

Site	Big Circle	Circle of Research (Survey)
Northern Devon	47 Community professionals and systems leaders who regularly engaged in Kailo initiated events	143 CYP and community members, responding to two surveys
Newham	10 Community professionals and systems leaders who regularly engaged in Kailo initiated events	44 CYP and community members responding to one survey

Across both sites other community professionals were also involved informally through ad hoc individual discussions and already established forums and local networks

Community professionals and systems leaders included local government professionals, local youth and community leaders and youth workers, health professionals, neurodiversity advocates, and charitable organisations [48]

Adapted from Santana de Lima et al., forthcoming

Engagement varied across sites. Big Circle participants were generally engaged, but not consistently enough to fully support the development of the co-designs or to build the level of buy-in needed for them to contribute resources and implement youth-generated strategies across all sites. In Northern Devon, sustained participation from community partners in one of the Small Circles enabled the strategy developed to be adopted immediately and prepared for implementation by a local community organisation. In the second Small Circle in the same site, and one Newham small circle, discussions are ongoing regarding the potential implementation of selected elements of the strategies, although engagement and progression took place more slowly in Newham. Across both sites, insights have been used and mentioned by different local organisations.

### Integration of group model building and research evidence

**Fig. 3:**
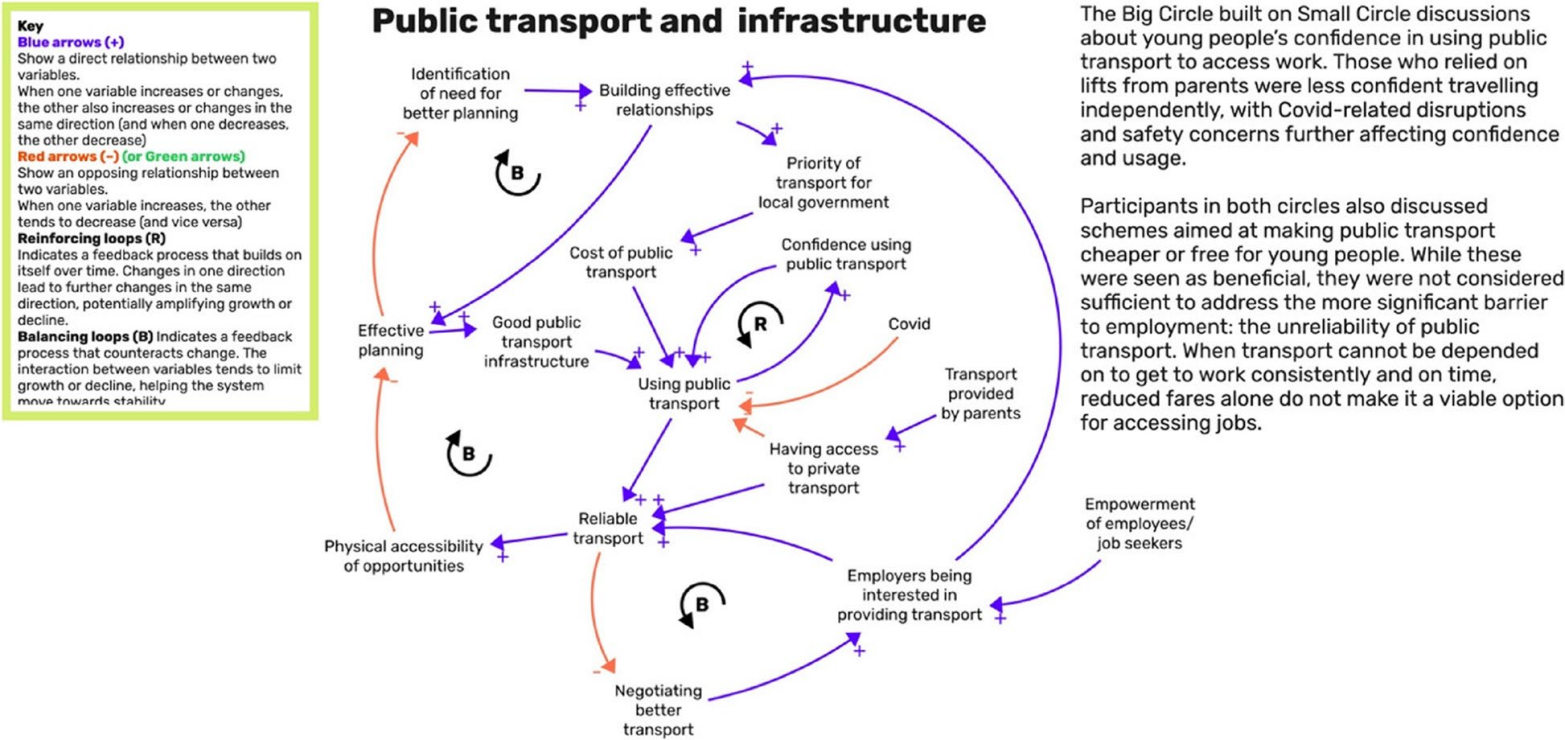
Northern Devon example of a causal loop diagram produced in GMB sessions

**Fig. 4:**
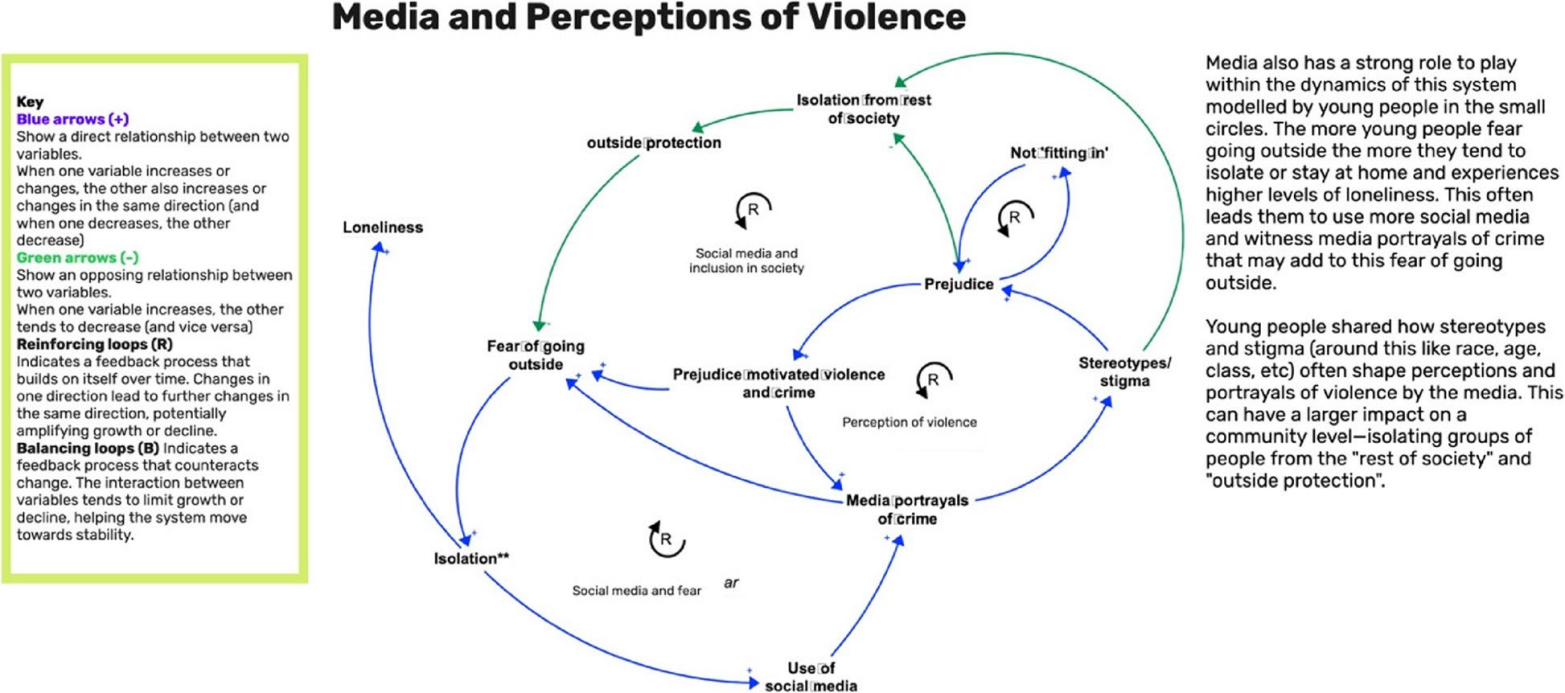
Newham example of a causal loop diagram produced in GMB sessions

Group Model Building was incorporated into the process to support the development of systems maps, which illustrated connections between variables related to issues identified across the Opportunity Areas [48] (see Figs. 3 and 4, and Systems Maps for Newham and Northern Devon).

Integration of research evidence proved more challenging to implement. More structured evidence-synthesis approaches, such as systematic review methods, were initially attempted but not used because the co-design questions evolved during the work, before systematic review processes could be completed. Rapid, pragmatic research summaries were used where relevant, although their production timelines did not always align with the scheduling of co-design sessions and stages of the process.

These factors influenced the degree to which existing research evidence informed the co-design process. Details of outputs produced through evidence integration are provided in Santana de Lima et al. [48].

### Resulting co-design strategies

A summary of the strategies developed through the Deeper Discovery and Co-Design process is provided in Tables 7 and 8.

**Table 7:** Summary of the resulting Northern Devon strategies from the co-design process [48]

Opportunity Area	What	Summary	Aims	Key Components
OA1: Building stronger informal community support networks to promote mental health awareness and literacy	*Neurodiversity and Mental Health Training and Resources: Barnstaple*	A programme to enhance understanding of mental health and neurodiversity among professionals, parents, and the wider community, aiming to reduce stigma and improve support for CYP	1. Reduce stigma around mental health and neurodiversity 2. Improve community-based support for CYP 3. Foster a more inclusive environment for addressing youth challenges	1. Tailored training sessions co-designed by individuals with lived experience 2. Campaigns and resources distributed online and in community spaces
OA2: Creating and enhancing access to more diverse opportunities for studies, employment, and recreation	*Careers Support Programme: Bideford*	A programme to introduce CYP to a wide range of career pathways and support them in achieving their goals, while building connections with local employers	1. Increase awareness of career options 2. Strengthen social capital and peer/employer networks 3. Provide training for employers to better engage with CYP 4. Align local employment systems with CYP’s evolving aspirations	1. Individual career guidance and personalised action plans 2. Work experience placements 3. Inspiring, skills-based sessions delivered by local employers

**Table 8:** Summary of the resulting Newham strategies from the co-design process [48]

Opportunity Area	What	Summary	Aims	Key Components
OA3: Reducing the impact of violence and crime and enhancing safety	*Life Skills Centre*	A dedicated centre offering workshops and courses designed to build CYP’s life skills, emotional resilience, and community connectedness	1. Empower CYP to make informed life choices 2. Improve mental health and wellbeing 3. Enhance future life prospects for those aged 16–25 (and younger via school-based programmes)	1. Free workshops on financial literacy, employability, cooking, and related topics 2. Youth-designed programmes to ensure cultural and contextual relevance. 3. Flexible, youth-friendly learning spaces led by experienced facilitators
OA4: Strengthening the role of local community infrastructure and activities for wellbeing	*Engagement Strategy for Youth Centres*	A strategy to raise awareness among school staff, parents, and CYP about the value of youth spaces and to reduce the stigma associated with youth centre attendance	1. Increase participation in youth clubs 2. Reduce stigma around youth centre use 3. Enable CYP to access community-based opportunities	1. TikTok© videos co-created with CYP to promote youth centres and normalise attendance 2. Youth workers embedded in schools to provide information, raise awareness, and offer taster sessions

In Northern Devon, co-designed strategies focused on supporting CYP in achieving their career goals and developing mental health and neurodiversity literacy training and materials. These initiatives aimed to raise awareness and improve the support available to CYP facing mental health challenges or who are neurodivergent, both in schools and within the home environment.

In Newham, the strategies included raising awareness about the benefits of youth centres and reducing the stigma associated with attending them, and the creation of a ‘Life Skills Centre’ to support CYP during transitional moments in their lives.

As illustrated above, co-design outputs varied across Small Circles and were related to CYP participants’ demographics. The Northern Devon Small Circle, which included more participants with neurodivergent needs, focused on mental health literacy and neurodiversity literacy. The Newham Small Circle, comprising older young people transitioning to adulthood, developed a strategy focused on life skills.

### Key themes shaping learning and reflection

The following themes support further discussion and reflection on how place-based co-design can enable systems change. In particular, they consider the extent to which this approach may create the conditions for improved adolescent mental health by addressing the social determinants of health through actionable, locally grounded strategies.

#### Place-based influences on adolescent mental health

##### Social determinants and lived experience

CYP and community members consistently emphasised that social determinants are experienced as deeply interconnected in their everyday lives. GMB was used to explore these relationships, helping participants uncover how various determinants interact within their local contexts (see Systems Maps for Newham and Northern Devon).

In Northern Devon, for example, Small Circle participants linked limited access to employment opportunities not only to a lack of job diversity but also to unreliable or non-existent transport networks (another key determinant), which limited access to employment opportunities and affected the availability of local opportunities (Fig. 3).

In Newham, discussions about violence and crime were closely tied to peer influence, social media (each also social determinants), and relative poverty, in complex ways (Fig. 4). Peers were associated with a sense of belonging and support, but also with potential exposure to violence and criminal behaviour. Social media was cited both as a factor increasing visibility (and resulting fear) of local episodes of violence, while also potentially protecting CYP by enabling ‘call for back-up' in threatening situations where CYP need immediate support. It also promoted desirability of material wealth, as did local ‘gentrification’ in parts of the borough.

These insights highlighted the complex and multifaceted nature of the challenges CYP experience in both areas and underscored the importance of designing responses that are tailored to the specific dynamics of each local context.

##### Similarities and differences: Focus on unemployment and the transition to adulthood

Despite differing OAs, CYP in both sites emphasised the transition to adulthood and access to employment as central themes in their co-design work. In Northern Devon, this was expected given that one of the prioritised OAs explicitly focused on unemployment. In Newham, while OA3 centred on violence and crime, participants identified access to opportunity (OA4) as an essential factor in preventing engagement in violence.

In Northern Devon, design outputs (see Systems Maps and Strategies developed) were focused on increasing knowledge, awareness, confidence, and motivation to explore different career opportunities, as well as enabling access to more diverse opportunities. Conversely, in Newham, the emphasis was on building life skills, employability skills, and financial literacy, such as budgeting and shopping on a limited income. These skills were seen as essential for reducing exposure to and protecting against experiences of violence.

These differences reflect the distinct local contexts shaping how unemployment is experienced. While the challenge in Northern Devon is primarily seen as a shortage of diverse opportunities, contributing to a ‘brain drain’ as CYP leave the region in search of education and employment, in Newham the issue is more often framed around intense competition for entry-level jobs, shaped by high living costs and limited access to roles that match CYPs needs and skills.

##### Similarities: The importance of youth spaces in mental health and wellbeing

Across both sites, CYP consistently emphasised the importance of youth spaces in supporting their mental health, wellbeing, and access to opportunities. In Newham, as illustrated in Table 5, this insight led to a co-designed strategy focused on raising awareness of the value of youth clubs. Youth spaces were framed as critical environments for nurturing wellbeing and fostering community. Similarly, in Northern Devon, youth spaces were recognised not only as safe and supportive environments but also as practical venues for delivering and implementing co-designed strategies.

This theme is particularly relevant to the goals of Kailo, as systems change involves not only designing new interventions but also identifying, sustaining, and enhancing what is already working within communities to support CYP’s mental health and wellbeing.

#### Designing systemic responses

##### Differences across OA’s: exploring ‘upstream’ challenges rather than ‘downstream’ solutions

Across both sites, the extent to which OAs addressed social determinants in systemic ways (i.e., rather than being individualised or focused on proximal interventions) varied. This variation was influenced by how issues were framed [47], who participated in the co-design process, and how the problem exploration and design of systems change response phases of the co-design process were implemented.

OAs that were clearly linked to a social determinant and did not presuppose a particular type of intervention (e.g. OA2) were more amenable to systemic exploration through GMB [70]. However, a recurrent challenge was the tendency to shift from systemic analysis toward more individualised, ‘downstream’ strategies during the transition from problem exploration to solution design.

In Northern Devon, the focus on unemployment (OA2, Table 4) was clear and bounded, which facilitated the exploration of related social determinants and systemic factors, as well as bringing clarity to the process of stakeholder engagement (e.g., schools, colleges and local government departments). The resulting strategy extends beyond a focus on CYP’s behaviours and attitudes to consider the roles of employers, the environments they foster, and the broader structures and systems that shape life in rural areas. Importantly, it also seeks to challenge prevailing societal beliefs regarding CYP’s future aspirations, particularly by reshaping perceptions of the types of employment deemed appropriate or attainable in rural contexts.

In contrast, OA1 (Table 4) in Northern Devon focused on mental health literacy and community support. This was less clearly defined in the Early Discovery phase and is shaped by multiple potential complex determinants, including discrimination, social environments, and peer relationships. This lack of definition made it harder to interrogate upstream factors in the Small Circle sessions, especially given the larger and more diverse group, which included significant numbers of both neurotypical and neurodivergent CYP. The resulting strategies were valuable but focused on attitudinal change and individual agency, with limited potential to shift the underlying systems of exclusion and stigma identified in the earlier systems mapping. However, the approach suggested aims to create the foundations to be able to do this in the future, via training and awareness campaigns.

A similar pattern was observed in Newham. OA3 (Table 5), which focused on violence and crime, was well defined and closely linked to the social determinant of exposure to violence. This gave the group a tangible issue to explore systemically and link to broader drivers such as inequality and marginalisation. However, the co-designed solutions remained largely focused on individual behaviours, encouraging CYP to adopt coping strategies or make use of supportive environments, rather than directly confronting the structural causes identified in the system map.

OA4 (Table 5) in Newham, which focused on promoting wellbeing through activities, reflected a more solution-led framing from the outset (i.e., activities for wellbeing). When explored from the perspectives of young people, this leaned towards a downstream orientation, making it difficult to surface or act on deeper contextual drivers. The emphasis on expanding access to wellbeing activities is important but with limited input from other stakeholders such as those commissioning and providing such activities, the promotional strategy again leaned towards awareness-raising among individual users rather than systemic change.

Across both sites, it is important to consider the role of GMB and the subsequent methods used in the co-design process. While systems mapping supported broad, interconnected understandings of CYP’s experiences and social determinants, the subsequent design methods and/or their framing defaulted to more individualised, programmatic responses [47]. It suggests that achieving systemic responses requires not only participatory design processes but also continued support to hold the focus on upstream levers and power structures, especially where there is a strong cultural or institutional pull towards service provision or behaviour-focused interventions.

Finally, this is not a critique of the ideas generated by the CYP (as they represent their insight as to what is important to address in their local area), but rather a challenge and limitation in meeting Kailo's objective of facilitating a process that engages CYP and other community partners to develop structural solutions addressing issues related to adolescent mental health in the wider population.

##### Differences: challenges in engaging the right people at the right time

Contextual factors shaped participation in the Deeper Discovery phase. In Northern Devon, CYP and community organisations were consistently engaged in Kailo activities and expressed a strong desire to improve their local area. There was also a shared recognition that initiatives like Kailo are rarely focused on rural southwest regions, which created a sense of opportunity and commitment.

In contrast, Newham presented a more saturated landscape in terms of initiatives targeting CYP’s lives [47]. Engagement beyond the Small Circles was generally lower, and fewer community partners had been involved in the Early Discovery phase. In addition, changes in the personnel delivering Kailo locally between the Early Discovery and Deeper Discovery phases created discontinuity, which disrupted essential community relationship-building and hindered the identification of needs [47].

Both sites experienced challenges in engaging the ‘right’ stakeholders in the Deeper Discovery Circles, Small and Big Circles. As the Deeper Discovery and Co-Design process was iterative by design, facilitators were initially uncertain about how each OA would evolve or narrow in scope. Without clearly defined proposals early on, it was difficult to generate alignment with the specific priorities or resource constraints of potential stakeholders [47]. This made it challenging to secure commitment to a systemic change effort that was still being shaped through co-design.

Involving system leaders in the Big Circle before strategies were clearly developed proved particularly difficult. However, in Northern Devon, once the co-designs were clearly defined and connected to specific sectors and relevant priorities, the systems leaders became more engaged because they recognised the relevance to their roles. Unfortunately, this alignment was not achieved in Newham, where system leaders remained more peripheral to the co-design process. Further exploration of these dynamics can be found in Allen et al. [47].

## Discussion

### Reflections and recommendations

In this section, we reflect on what helped and hindered a focus on addressing the social determinants of CYP’s mental health and wellbeing through the Deeper Discovery and Co-design process. Drawing from this experience, we also offer recommendations for how future research and design initiatives might create and maintain this focus more effectively.

#### What helped and hindered a focus on designing systems change responses to the social determinants of CYP’s mental health?

##### Clarity and framing of place-based social determinants

Across both sites, OAs that were clearly framed around a specific social determinant, and that remained agnostic to predefined solutions, proved more conducive to exploration through a systems lens. These areas allowed for more effective use of GMB, which in turn helped to identify systemic intervention points and foster shared goals among participants [47]. Furthermore, the place-based nature of the inquiry enabled exploration of these determinants in locally specific terms, increasing their salience and relevance for participants [71], as was evident in the Deeper Discovery Circles.

##### Blending co-design with complementary methods

Integrating co-design with complementary approaches highlighted the complexity of how CYP experience the social determinants identified within their prioritised OAs. As noted in related studies [72], GMB is particularly effective in illustrating interconnections among multiple, interacting influences. In this context, GMB enabled participants across all Deeper Discovery Circles to engage in cross-cutting dialogue, transcending thematic or professional silos. It helped build a shared understanding of how specific social determinants operate and influence local mental health outcomes, thus supporting a sustained focus on broader contextual drivers [49].

However, this approach had its limitations. Creating co-designs that genuinely support system-level change, rather than individual-level responses, would have required further use of methods and approaches grounded in systems change, applied consistently during both the problem exploration and the design of practical responses to address social determinants.

This bias toward individual-focused solutions is mirrored in the existing evidence base [47], which often prioritises individual-level explanations and interventions over community or systemic approaches [73, 74]. Consequently, literature’s contribution to context-specific understanding could be said to be limited in some cases, in part due to its insufficient attention to social determinants and place-based system change [75].

##### The crucial value and limits of lived experience

CYP contributed rich insights throughout the co-design process, offering contextualised and nuanced understandings of local challenges affecting youth mental health. This echoes findings from prior research [76–78], which highlight the value of lived experience in enhancing the depth and relevance of participatory processes.

However, due to their position, knowledge, and lived experience, CYP were not always equipped, or best positioned to identify *how* broader social determinants could be structurally addressed beyond an individually focused or service-level response. As Conway-Moore et al. [79] note, participatory processes often generate ideas rooted in participants’ immediate environments and experiences, as these are more concrete and accessible. While these ideas are critical, they may be insufficient on their own to promote systems change without additional structures or supports that bridge the gap between lived experience and institutional or policy reform.

##### Stakeholders’ involvement

The exploratory nature of the co-design process enabled participants to progressively develop a shared mission [47]. However, this same openness and uncertainty, which is required for adaptive co-design [80], combined with the time-intensive nature of the approach [81], are frequently cited as aspects that can present challenges for stakeholder engagement [55]. In particular, these were not always aligned with the strategic and operational priorities, capacity, and resource constraints of some stakeholders, which limited their ability to participate consistently, or at all [48].

Inconsistent participation across the Deeper Discovery Circles restricted the diversity of perspectives informing the system maps and reduced the capacity to identify and act upon the structural levers needed to address social determinants meaningfully. This issue is also well recognised in the wider literature on participatory and systems approaches [82].

These challenges underscore the importance of considering when, where, and how the expertise of CYP, community members, and professionals can be most effectively combined in co-design processes aiming for systems change.

#### Recommendations for co-designing systems change responses to address the social determinants of mental health

##### Consistently emphasise the social determinants

*OAs* can be more consistently framed to focus on social determinants of mental health, rather than mental health itself. Practical tools and resources for use in the Deeper Discovery and Co-design Circles, and beyond the Small Circles, can incorporate a clear and consistent definition or framework for the social determinants, and the nature of systems change, which could be shared across engagement circles.

Co-design is messy [56, 83], and sometimes priorities might shift in complex ways. Thus, communication and storytelling play a crucial role in maintaining a consistent narrative about the work (one that clearly defines its boundaries, particularly in relation to social determinants and systems change) and reinforces them throughout each stage of the co-design process and across both the Deeper Discovery and Co-design Circles [47].

##### Prioritise the voices of CYP alongside those who are committed to supporting them

The intention to prioritise and centre youth voices, whilst essential, needs to be coupled with the adequate involvement of wider groups of relevant stakeholders within a particular community or system, who have an important role to play in supporting CYP locally [47]. Systems change requires not only the articulation of young people’s priorities, but also the translation of these priorities into changes to policies, processes and practices that are largely shaped within adult-led systems [48].

In the Small Circles, further input was required from professionals with experience of these adult-led systems, including their policy frameworks, implementation opportunities and constraints, operational constraints and high-impact levers [48]. This expertise could have supported young people to better understand how their desired changes might be realised in practice. Greater involvement of community professionals throughout all Small Circle sessions, rather than at discrete points, may have strengthened this, including local government professionals, youth workers, community organisation staff, health professionals and parents.

Furthermore, the demographics of the young people co-designing require careful consideration. As expected, the reach of strategies will likely reflect the characteristics of the young people who designed them [48, 79], as seen with one of the strategies at one of the Small Circles in each site.

##### Enable joined-up action across sectors and levels in the local system

Addressing the social determinants of health and wellbeing through a systems lens requires systems leaders and community professionals to actively explore and understand the interdependencies between issues, and the implications this has for coordinated action [84]*.* Systems change cannot be achieved through isolated interventions; it depends on alignment across policy departments [85–87], sectors and levels of governance [88].

This requires more than acknowledging complexity. It involves establishing appropriate governance, oversight and coordination arrangements, and creating structured opportunities for key stakeholders to engage at critical points throughout the discovery and co-design process, to lay the groundwork for later adoption and implementation. Closely related is the need to involve those who hold decision-making power over resources [89]. Local commissioners, policymakers and senior practitioners bring insight into the structures, policies and funding mechanisms that can enable and constrain the implementation of co-designed solutions, as well as the authority required to support sustained change.

In practice, this could have been strengthened by engaging Big Circle stakeholders earlier and more consistently in the development of the Small Circles. Establishing the Big Circles in advance may have helped build shared understanding of the programme’s aims, develop trust in the process through early dialogue, and foster reciprocity by clarifying what support Small Circles would need and the nature of stakeholder involvement required. More structured, regular and flexible engagement opportunities may also have improved buy-in. The importance of involving a diverse range of stakeholders in this way is well supported in the literature and is considered crucial for developing structural solutions [89].

##### Acknowledging the different priorities of diverse groups involved

The Kailo team encountered familiar challenges in navigating the differing priorities, interests and levels of influence held by young people, community partners, systems leaders and the research team itself. *A key learning is the importance of making the incentives, expectations, roles and decision-making authority of each stakeholder group explicit from the outset, rather than assuming alignment.*

For co-design to support systems change, these differences must be understood and explored throughout the process, with deliberate attention to how contributions are balanced and whose priorities shape key decisions at different stages. This includes recognising where decisions are being made and ensuring transparency about constraints within the system [89].

Research and design teams also need to remain open and responsive, adapting their approach considering co-designers’ feedback, emerging needs and the overarching aims of the work. This reflexivity is essential to maintaining trust, legitimacy and momentum across diverse groups with unequal power and influence [90].

##### Incorporate frameworks that support integration of systems change into the co-design process

*While co-design alone, and particularly Small Circles of co-design, will not generate all the answers to complex challenges, it plays a crucial role within the Kailo Framework.* To effectively address these challenges, co-design must be combined with approaches that enable systems change analysis and considerations. In Kailo, this was initially done through the integration of GMB, and systems mapping into the co-design methodology, as well as considerations of the six conditions of systems change to understand where change could be effective and impactful. However, other and more flexible approaches could also be explored, including systems icebergs [91], network analysis [92] and root cause analysis [93]. This integration helps translate complex ideas and systemic perspectives into practical tools that support exploring and developing strategies [89]. Crucially, this work requires a strong commitment to transforming the structures that create inequalities and recognising how some individually focused interventions may inadvertently cause or reinforce those inequalities [94, 95]. This is particularly relevant for those who may also be focused on behaviour changes [95]. While public health policies often emphasise social justice, they have historically tended to default to encouraging individual behaviour change in an approach known as "lifestyle drift" [96]. Though not inherently wrong, this focus alone is insufficient and can unintentionally reinforce existing inequalities [97].

## Conclusion

This paper has reflected on learning from the Kailo Deeper Discovery process, which focused on co-designing systemic strategies to address the social determinants of CYP’s mental health and wellbeing. The co-design approach adopted was centred around CYP’s participation and enabled community members (CYP and others) to explore various ideas and potential strategies. The strategies developed all represent ways to contribute to addressing the issues raised.

There were important challenges associated with navigating the pull towards individual and services approaches and solutions while attempting to develop co-designs to address mental health issues through the social determinants and a systemic lens. This suggests careful consideration is required around where, when and how different groups of CYP with lived experience, as well as other groups of community members, are best placed to engage in the development of solutions to issues they are currently experiencing, as well as what supports are required. This is particularly important in community participation efforts exploring complex issues targeting upstream mental health and wellbeing challenges and their associated social determinants.

Key recommendations included the need to consistently centre social determinants when exploring and designing strategies; to explicitly acknowledge and address the limitations of individualised approaches; and to recognise the important role that different community members and wider stakeholders play (e.g., CYP, community members, professional stakeholders) in both exploring issues and actively contributing to the development of solutions.

These insights are directly informing subsequent adaptations of the Kailo framework and design, contributing to the wider literature on how and where co-design works to lead or support the development of solutions to issues faced by CYP and other community members.

## Data Availability

The data shall be made available upon request. Please contact the corresponding author Ediane Santana_de_Lima@dartington.org.uk.

## Abbreviations

CYP: Children and Young People
SIG: Social Innovation Generation
OAs: Opportunity Areas’
GMB: Group Model Building
